# Examining pancreatic stone protein response in ICU-acquired bloodstream infections: a matched event analysis

**DOI:** 10.1186/s40635-024-00634-7

**Published:** 2024-05-28

**Authors:** Diede Verlaan, Lennie P. G. Derde, Tom van der Poll, Marc J. M. Bonten, Olaf L. Cremer

**Affiliations:** 1 Department of Intensive Care Medicine, University Medical Centre Utrecht, Utrecht University, Heidelberglaan 100, F06.149, P.O. Box 85500, 3508 GA Utrecht, The Netherlands; 2 Julius Centre for Health Sciences and Primary Care, University Medical Centre Utrecht, Utrecht University, Utrecht, The Netherlands; 3 Centre of Experimental and Molecular Medicine & Division of Infectious Diseases, Amsterdam University Medical Centres, Location AMC, University of Amsterdam, Amsterdam, The Netherlands

**Keywords:** Pancreatic stone protein, PSP, Bloodstream infection, Biomarker

## Abstract

**Background:**

Pancreatic stone protein (PSP) exhibits potential as a plasma biomarker for infection diagnosis and risk stratification in critically ill patients, but its significance in nosocomial infection and intensive care unit (ICU)-acquired bloodstream infection (BSI) remains unclear. This study explores the temporal responses of PSP in ICU-acquired BSI caused by different pathogens.

**Methods:**

From a large cohort of ICU patients, we selected episodes of ICU-acquired BSI caused by Gram-negative rods (GNRs), enterococci, or *Candida* species. Events were matched on length of ICU stay at infection onset, Severe Organ Failure Assessment (SOFA) score, presence of immune deficiency, and use of renal replacement therapy. PSP concentrations were measured at infection onset (T0) and at 24, 48 and 72 h prior to infection onset as defined by the first occurrence of a positive blood culture. Absolute and trend differences in PSP levels between pathogen groups were analysed using one-way analysis of variance. Sensitivity analyses were performed in events with a new or worsening systematic inflammatory response based on C-reactive protein, white cell count and fever.

**Results:**

We analysed 30 BSI cases per pathogen group. Median (IQR) BSI onset was on day 9 (6–12). Overall, PSP levels were high (381 (237–539) ng/ml), with 18% of values exceeding the assay’s measurement range. Across all 90 BSI cases, there was no clear trend over time (median change 34 (− 75–189) ng/ml from T-72 to T0). PSP concentrations at infection onset were 406 (229–497), 350 (223–608), and 480 (327–965) ng/ml, for GNR, enterococci, and *Candida* species, respectively (*p* = 0.32). At every time point, absolute PSP levels and trends did not differ significantly between pathogens. PSP values at T0 correlated with SOFA scores. Eighteen (20%) of 90 BSI events did not exhibit a systemic inflammatory response, primarily in *Candida* species. No clear change in PSP concentration before BSI onset or between-group differences were found in sensitivity analyses of 72 cases.

**Conclusions:**

Against a background of overall (very) high plasma PSP levels in critically ill patients, we did not find clear temporal patterns or any pathogen-specific differences in PSP response in the days preceding onset of ICU-acquired BSI.

## Background

Pancreatic stone protein (PSP) is an emerging plasma biomarker that may have utility in the early diagnosis of sepsis [1, 2]. In several prospective cohort studies, conducted both in emergency room and intensive care unit (ICU) settings, the diagnostic performance of PSP in discriminating sepsis from uncomplicated infection and/or sterile inflammation appeared to be at least comparable—and perhaps superior—to other commonly used biomarkers, such as C-reactive protein (CRP) and procalcitonin [3–5]. Apart from use as a diagnostic test, higher PSP concentrations were also associated with higher disease severity and worse outcomes in patients with sepsis and septic shock [6, 7].

Initial observations of PSP secretion by the pancreas in response to (remote) organ damage and sepsis have led to the consideration of this biomarker as an indicator of systemic stress [8, 9]. PSP is a C-type lectin-binding protein, a class of molecules that plays an important role in various biological processes such as pathogen recognition and signalling receptors in innate and adaptive immunity and is essential in inflammatory responses [10, 11]. Through upregulation by interleukin-6 and other cytokines released during inflammation, PSP release promotes proinflammatory activity by recruiting immune cells to sites of infection or inflammation [12, 13]. Importantly, these processes occur very early during the immune response. Observations of rising PSP concentrations even as early as 5 days prior to a clinical diagnosis of sepsis have raised hope that PSP could serve as a presymptomatic biomarker of sepsis [3].

Previous studies on the diagnostic performance of PSP mostly focused on community-acquired sepsis presentations in the emergency room, the detection of sepsis at the time of admission to intensive or high-dependency care, and specific use cases, such as identifying sepsis secondary to inhalation injury in burn patients [4, 14, 15]. However, the experience with this biomarker for the early diagnosis of nosocomial infections in an unselected ICU population is limited. Such infections are more often caused by endogenous organisms, which are bacteria that the host is already colonized with. These include Gram-negative *Enterobacteriaceae* such as *Escherichia coli*, *Klebsiella pneumoniae* and *Pseudomonas aeruginosa*, along with Gram-positive bacteria such as enterococci and coagulase-negative staphylococci of lower—yet significant—pathogenicity [16]. ICU patients, particularly those with impaired immunity, may also be more susceptible to other opportunistic infections such as *Candida* species [17].

Overall, PSP trajectories in these types of infections have not been studied well, particularly the extent to which the host response differs for various pathogens. Therefore, we explored temporal PSP responses in critically ill patients who developed ICU-acquired bloodstream infection (BSI) caused by either Gram-negative rods (GNR), enterococci, or *Candida* species and evaluated if PSP responses differed between pathogen types.

## Methods

### Study design

The present study was conducted using a nested matched-case design. From the Molecular Diagnosis and Risk Stratification of Sepsis (MARS) cohort, we selected ICU-acquired bloodstream infections caused by either one of the following three pathogen groups of interest: (1) GNR (including *E. coli*; *K. pneumoniae*; *P. aeruginosa*; *Aeromonas*; *M. morganii*; *S. marcescens*; *S. maltophilia*; *H. influenzae*; *P. mirabilis*; *P. vulgaris*), (2) enterococcal species (*E. Faecalis*; *E. faecium*), and (3) *Candida* species (*C. albicans*; *C. glabrata*; *C. lusitaniae*; *C. krusei*; *C. parapilosis or C. species* not further determined).

Events were considered to be ICU acquired if the following criteria were met: (1) a first occurrence of a positive blood culture at least 96 h after ICU admission and (2) no prior blood culture yielding the same pathogen for at least 30 days. Blood cultures yielding more than one pathogen were excluded. To avoid the selection of possible contaminants, positive cultures with an incubation period of > 72 h were ineligible for selection. Furthermore, enterococci were included only if enterococcal species had been isolated from at least two consecutive blood cultures taken on different days (whereas all GNR and *Candida* species isolated from blood cultures were considered true positives). Final exclusion criteria included abdominal surgery in the week prior to infection onset and the presence of chronic or acute pancreatitis, as these conditions may directly affect the reliability of the PSP biomarker [8].

Subsequently, we conducted a matched case analysis. For the matching procedure, available BSI cases due to *Candida* species were matched to individual BSI cases from the (larger) GNR group in a 1:1 ratio. Next, the resulting pairs were also matched to cases from the (largest) enterococci group. Matching criteria were (1) the presence of immunodeficiency at ICU admission; (2) length of ICU stay on the day of BSI onset (± 4 days), (3) use of renal replacement therapy 72 h before BSI onset, and (4) Sequential Organ Failure Score (SOFA) (± 4 points) 72 h before BSI onset. To this end, immunodeficiency was defined as the active use of immunosuppressive drugs upon ICU admission (including chronic or high-dose corticosteroids, calcineurin inhibitors, mycophenolate mofetil, and others), hematologic malignancy, active cancer (including chemotherapy or radiotherapy in the year prior to admission), neutropenia, or any other documented humoral or cellular deficiency. Renal replacement therapy was used as a matching criterion, as kidney dysfunction may directly affect PSP levels [18, 19].

### Biomarker measurement

We used consecutive daily samples that had been collected as part of the MARS cohort during three days prior to infection onset (i.e., the day on which a first positive blood culture was drawn). EDTA blood was spun at 1500 rpm for 15 min and supernatant plasma was stored at − 80 °C within a maximum of 4 h from sampling. PSP was subsequently measured using the CE-marked in-vitro diagnostic PSP capsule on the abioSCOPE® platform (Abionic SA, Epalinges, Switzerland). This ‘point-of care’ device measures PSP concentrations in plasma between 20 and 600 ng/mL within 8 min using nanofluidic immunoassay technology [20]. Samples yielding out-of-range values were diluted 1:4 and subsequently retested to achieve an accurate result.

### Patient population

We exploited the MARS cohort, in which consecutive critically ill patients with an expected ICU length of stay > 48 h had been prospectively enrolled in two tertiary mixed ICUs in The Netherlands. Participating centres were the University Medical Centre Utrecht (recruiting 2011–2019) and the Amsterdam University Medical Centre (recruiting 2011–2013). Selective digestive decontamination was part of standard care in both centres [21]. The MARS project was approved by the Medical Ethics Committees of both participating hospitals (protocol number 10-056). The current study was additionally reviewed by the UMCU Biobank Research Ethics Committee (protocol number 21-405).

### Statistical analysis

Differences in patient and event characteristics were analysed using one-way analysis of variance (ANOVA), Kruskal–Wallis tests, or chi-square tests, as appropriate. To facilitate the analysis of temporal trends, a delta PSP value was calculated for each BSI case reflecting the absolute change from 72 h before infection (T-72) to infection onset (T0). Absolute and trend differences in PSP levels between groups were subsequently analysed using one-way ANOVA. For the latter analysis, all PSP values were log-transformed.

To gain detailed insight into how PSP responds to BSI onset, we used several explorative approaches, some of which were informed by post hoc findings. First, we investigated the concomitant rise and fall of other inflammatory markers, including CRP, white blood cell count, and fever. To this end, we defined a systemic inflammatory response as the occurrence of at least one of the following criteria in the 72 h preceding onset of infection: (1) a CRP ≥ 100 mg/L with at least a 20 mg/L increase compared to the previous day, (2) a white blood cell count ≥ 12 × 10^9^/L with at least a 1 × 10^9^/L increase compared to the previous day, (3) a white blood cell count ≤ 4 × 10^9^/L with at least a 1 × 10^9^/L decrease compared to the previous day, or (4) fever, defined as a temperature ≥ 38.3 °C with at least a 1 °C rise compared to the previous day. We then conducted a sensitivity analysis excluding BSI events that did not meet any of these criteria to investigate our hypothesis that patients lacking a systemic inflammatory response might be distorting the temporal trend. Second, since blood cultures were not systematically obtained on a daily basis, it is possible that pathogens were in fact already present in the bloodstream for some time before detection. To address this issue, we realigned the timelines of 72 cases of patients with a systemic inflammatory response by assuming that T0 (i.e., the true onset of BSI) coincided with the observed peak in CRP level if this peak occurred before a first positive culture. This is a conservative approach since CRP levels may take up to 50 h to reach their maximum in simple infection [22]. However, we assumed that a rise in CRP (in the absence of new surgical trauma) could at least approximate potential diagnostic delays. Finally, we constructed subgroups that were stratified by severity of disease (SOFA-score of 7 or higher) as measured on the day of BSI onset. A Wilcoxon rank sum test was then used to assess differences between groups.

We used R Studio 2023.12.1 Build 402 (R foundation for Statistical Computing, Vienna, Austria) for all statistical analyses. Continuous variables are presented as medians with interquartile ranges (IQRs). Discrete values are shown as counts with percentages. All reported p values are two-sided, and statistical significance was set at an *α*-value of 0.05.

## Results

### Patient and event characteristics

**Fig. 1:**
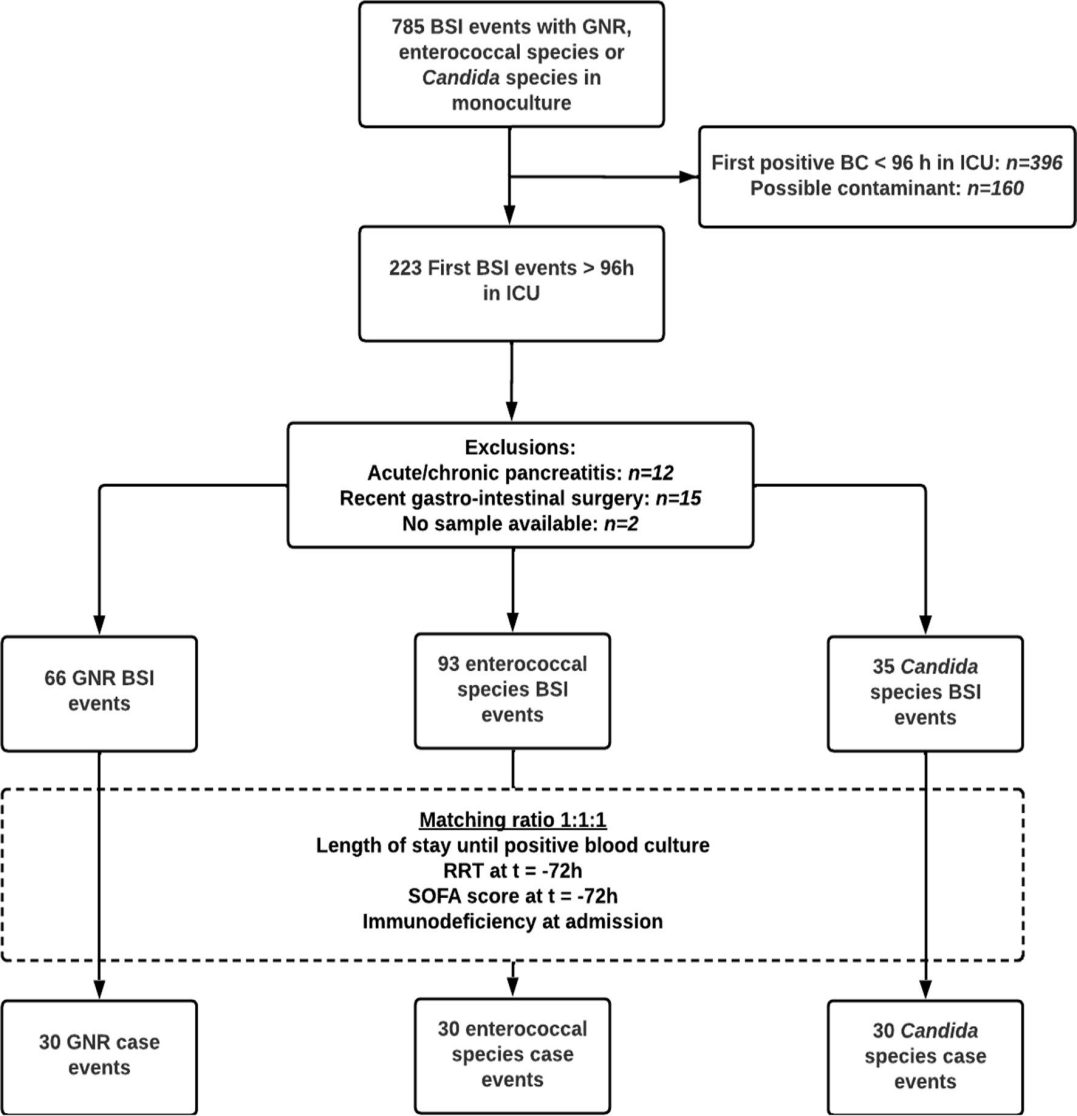
Event inclusion. *BSI* bloodstream infection, *GNR* gram negative rods, *BC* blood culture, *ICU* intensive care unit, *RRT* renal replacement therapy, *SOFA* Sequential Organ Failure Assessment

Among 8533 patients in the MARS cohort (contributing a total of 82,734 observation days in the ICU), there were 223 episodes of monoculture ICU-acquired BSI due to either GNR, enterococci, or *Candida* species in 210 patients, yielding an estimated incidence rate of 0.8 (95% CI 0.6–1.0), 1.4 (95% CI 1.1–1.6), and 0.5 (95% CI 0.4–6.9) events per 1000 patient-days at risk for each pathogen, respectively (Fig. 1). After excluding events associated with abdominal surgery in the week prior to infection onset and the presence of chronic or acute pancreatitis 194 cases of BSI remained: 66 due to GNR, 93 due to enterococcal species, and 35 due to *Candida* species. Across these three pathogen groups, 69 (i.e., 23 sets of three) BSI events could be fully matched on all criteria, whereas 21 events (seven sets) were randomly selected and added to each group to make a total of 30 cases per pathogen type originating from 87 patients.

Matching resulted in three patient groups with comparable comorbidity status, admission characteristics, and disease severity as measured by SOFA score (Table 1). The median (IQR) time of BSI infection onset was day 9 (6–12) in the ICU, which was similar across groups. However, patients with BSI due to *Candida* species were younger (*p* = 0.03) and had lower use of indwelling central venous catheters (*p* = 0.03), whereas patients with enterococcal infections were more often male (*p* = 0.02). The median time to blood culture positivity for the included events was 18 (13–28), 12 (10–13) and 51 (18–84) h for GNR, enterococci and *Candida* species, respectively. However, please note that the apparent fast growth of enterococci was entirely due to strict selection criteria imposed by us to prevent the selection of contaminated blood draws. Following BSI diagnosis, antimicrobial treatment was started in 82 (91%) of 90 cases for a median duration of 9 (3–19) days; six patients died before the culture results were known and in two patients with enterococcal BSI indwelling catheters were removed without additional treatment.

**Table 1:** Demographics, comorbidities, and clinical characteristics of bloodstream infection events by pathogen type

Variables	Gram negative rods (*n* = 30)	Enterococcal species (*n* = 30)	*Candida* species (*n* = 30)	*p*-value
Patient characteristics				
Age (years)	62 (54–70)	65 (59–72)	52 (47–67)	0.03
Sex (male)	18 (60)	26 (87)	17 (57)	0.02
Diabetes Mellitus	9 (30)	4 (13)	3 (10)	0.09
Solid or hematologic malignancies	7 (23)	7 (23)	10 (33)	0.60
Immunodeficiency	7 (23)	11 (37)	9 (30)	0.53
Charlson Comorbidity Index	2 (0–2)	1 (0–3)	2 (0–2)	0.78
ICU admission characteristics				
Surgical reason for admission	16 (53)	13 (43)	12 (40)	0.83
Prior ICU admission^1^	6 (20)	8 (27)	7 (23)	0.83
APACHE IV score	93 (73–119)	89 (77–106)	86 (67–107)	0.55
ICU mortality	17 (57)	14 (47)	11 (37)	0.30
Clinical characteristics at BSI *onset*^*2*^				
Days in ICU	9 (6–13)	9 (7–11)	8 (4–11)	0.21
SOFA score	8 (5–12)	9 (6–12)	9 (6–13)	0.95
Renal replacement therapy	15 (50)	15 (50)	13 (43)	0.84
Catecholamine use	10 (33)	11 (37)	7 (23)	0.51
Prior antibiotic exposure during ICU stay	24 (80)	28 (93)	22 (73)	0.23
Indwelling arterial catheter	28 (93)	26 (87)	28 (93)	0.58
Indwelling central venous catheter	28 (93)	28 (93)	22 (73)	0.03
Presumed source of BSI				
CRBSI	7 (23)	19 (63)	10 (33)	0.004
Cardiac	2 (7)	0 (0)	2 (7)	0.35
Intra-abdominal	0 (0)	2 (10)	0 (0)	0.13
Surgical site^3^	7 (23)	1 (0)	1 (3)	0.01
BSI without primary focus	9 (30)	8 (27)	13 (43)	0.35
Other	5 (17)	0 (0)	4 (13)	0.07

1. Prior ICU admission signifies ICU admission within the same hospital stay 2. Treatments and disease characteristics represent the clinical situation immediately prior to BSI onset 3. Any infection originating in surgical wounds or the organs/spaces opened or manipulated during an operative procedure. Data are presented as absolute frequencies (column percentages) or medians (IQR). *P*-values reflect chi-squared, ANOVA or Kruskal–Wallis tests, as appropriate

*APACHE* Acute Physiology and Chronic Health Evaluation, *BC* Blood culture, *BSI* Bloodstream infection, *CRBSI* Catheter-related bloodstream infection, *ICU* intensive care unit, *SOFA* Sequential Organ Failure Assessment

### Temporal patterns in PSP concentration

**Fig. 2:**
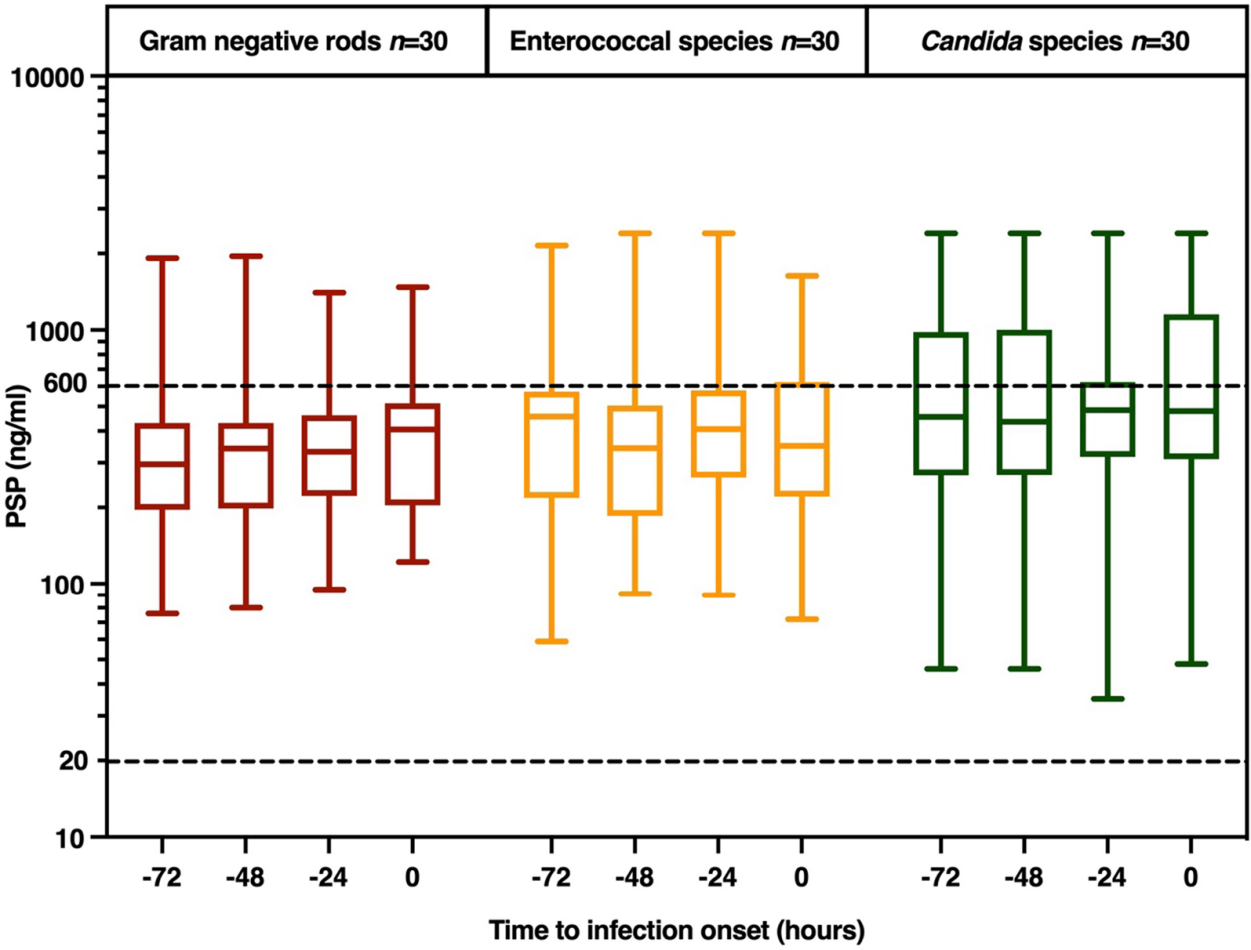
PSP plasma concentrations by pathogen type in the days preceding BSI onset. Boxplots show medians with interquartile range. The dotted lines indicate the upper (600 ng/ml) and lower (20 ng/ml) limits of the detection range. *PSP* pancreatic stone protein, *BSI* bloodstream infection

We observed high median (IQR) plasma PSP levels (381 (237–538) ng/ml), with 18% of all samples (*n* = 349) yielding values above the manufacturer-specified measurement range for the assay. Overall, the measured values were 5 (3–7) times higher than the reported mean concentration [83 (95% CI 78–88) ng/ml] in 211 healthy adults, as observed in the clinical trial report from Abionic (ClinicalTrials.gov identifiers: NCT05849935 and NCT04409561). Plasma PSP levels at T-72 were 296 (200–424), 456 (237–566), and 455 (290–847) ng/ml in the GNR, enterococcal, and *Candida* BSI groups (*p* = 0.16), and these were 406 (229–497) ng/ml, 350 (223–608) ng/ml, and 480 (327–965) ng/ml (*p* = 0.32), respectively, at infection onset (T0). No statistically significant between-group differences at any (other) time point were observed, although absolute PSP levels tended to be lower in GNR compared to *Candida* infections (Fig. 2). Overall, there was no clear trend over time in the days before BSI onset (*p* = 0.15), whereas for individual patients the observed change in PSP concentration from T-72 to T0 hours was 49 (− 63–211), 32 (− 60–109), and 31 (− 191–120) ng/ml (*p* = 0.82), for the GNR, enterococcal, and *Candida* BSI groups, respectively.

### Sensitivity analyses

Based on post hoc definitions, 72 (80%) of the 90 BSI events were associated with a clinically apparent new systemic inflammatory response. Restriction analyses of these episodes also failed to identify clear trends in PSP concentrations over time (*p* = 0.15) or statistically significant differences between the three causal pathogen groups (*p* = 0.77; Table 2). Among the 18 (20%) events without a new systemic inflammatory response, the rate of ‘indolent’ infection onset differed per causal pathogen group: 3 (10%) for GNR, 5 (17%) for enterococci and 10 (33%) for *Candida* species.

**Table 2:** Plasma PSP concentrations (ng/ml) by pathogen type in BSI patients who develop a systemic inflammatory response (*n* = 72)

Marker	Gram negative rods (*n* = 27)	Enterococcal species (*n* = 25)	*Candida* species (*n* = 20)	*p*-value
PSP *t* = − 72	300 (194–423)	465 (236–571)	489 (282–1017)	0.10
PSP *t* = − 48	319 (192–378)	348 (224–475)	482 (279–1017)	0.09
PSP *t* = − 24	336 (218–499)	407 (286–592)	544 (308–636)	0.16
PSP *t* = 0	400 (251–524)	481 (235–708)	480 (357–650)	0.53
∆ PSP^a^	50 (− 22–208)	33 (− 48–164)	36 (− 167–151)	0.77

Plasma PSP levels are presented as median with interquartile range. Plasma PSP levels were natural log-transformed for ANOVA test results

*BSI* bloodstream infection, *PSP* pancreatic stone protein

^a^This delta measure reflects the within-event difference from T −72 h to onset of BSI (T0)

**Fig. 3:**
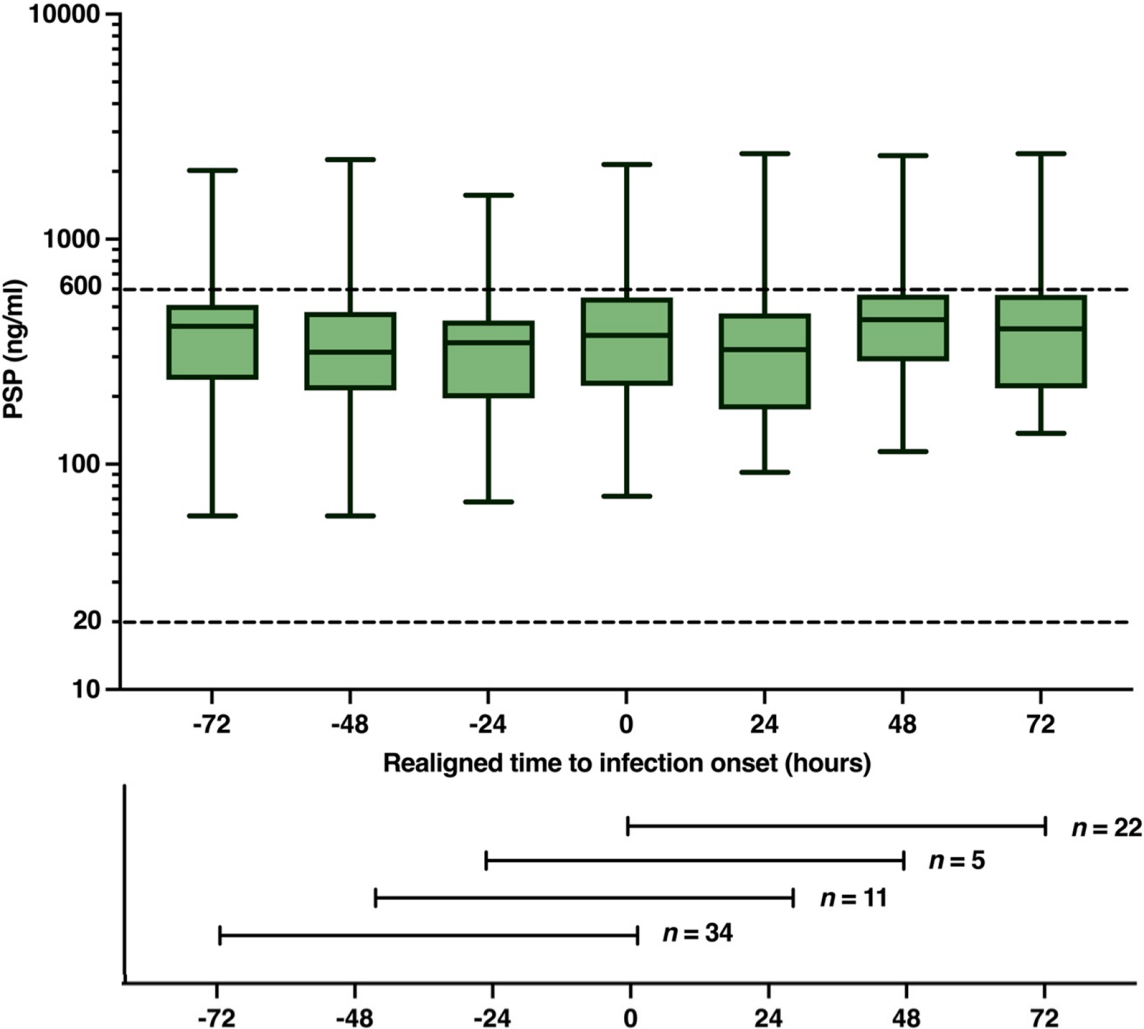
Plasma PSP concentrations for all BSI events (*n* = 72) after re-alignment according to the day of maximum CRP response. The time shifting aimed to adjust for potential delays in BSI diagnosis and was only performed if a CRP peak had occurred prior to BSI onset (by a first positive blood culture). Plasma PSP levels from T −72 h to T +72 h after presumed onset are presented. In 34/72 events peak CRP aligned with BSI onset (T0). *Boxplots* show medians with interquartile range. The dotted lines indicate the upper (600 ng/ml) and lower (20 ng/ml) limits of the detection range. *PSP* pancreatic stone protein, *BSI* bloodstream infection

In a second sensitivity analysis, to correct for diagnostic delays in BSI detection, we observed that in 38/72 (53%) BSI events, CRP peaked prior to T0, indicating a potential error in the timing of BSI onset of ≥ 24 h for 11 (15%), ≥ 48 h for 5 (7%), and ≥ 72 h for 22 (31%) cases. Realignment of PSP measurements according to the reconstructed 'true' date of BSI (which was estimated to coincide with or precede the CRP peak) did not reveal clear temporal patterns across all groups combined (Fig. 3) (*p* = 0.58).

Finally, we stratified subgroups of cases upon severity of disease at BSI onset regardless of causative pathogen type. Median PSP plasma values were higher in patients suffering from higher disease severity [171 (127–329) versus 482 (327–680) ng/ml] for patients with SOFA < 7 versus ≥ 7 (*p* < 0.001). Similar patterns were observed in all three causative pathogen groups (Table 3).

**Table 3:** PSP concentrations upon BSI onset stratified by pathogen type and severity of disease

Stratified analysis	n	Low severity of disease	n	High severity of disease	*p*-value
All	21	171 (127–329)	66	482 (327–680)	< 0.001
Gram-negative rods	8	181 (129–409)	21	415 (323–540)	0.030
Enterococcal species	5	149 (92–220)	23	500 (268–708)	0.002
*Candida* species	8	196 (146–341)	22	520 (438–1345)	0.005

Plasma PSP levels are presented as medians with interquartile ranges (Q1–Q3). Severity of disease was stratified by a SOFA-score of 7 or higher, test results are presented according to Wilcoxon rank sum test

*BSI* bloodstream infection, *PSP* pancreatic stone protein, *SOFA* Sequential Organ failure Assessment

## Discussion

We measured PSP levels in plasma in the days preceding ICU-acquired BSI caused by either GNR, enterococci, or *Candida* species and assessed differences in PSP response between pathogen types. Overall, we observed high PSP concentrations at all time points for all causative pathogens, with values typically 3–7 times higher than the reported normal value and above the detection range of the assay in 18% of measurements. In addition, we did not observe a clear, unambiguous pattern in PSP response over time, either for the combined events or when analysing causative pathogens separately. This may limit the utility of PSP as a diagnostic marker of nosocomial BSI in critically ill patients.

Our findings differ from those reported in previous studies. In a study involving an unselected population of patients undergoing cardiac surgery PSP levels increased faster in the initial three days of ICU admission among patients who developed ICU-acquired infections compared to those with an uneventful course. This difference could potentially be attributed to the timing of PSP measurements, as this study primarily focused on the early stages of ICU admission. In contrast, our study specifically centred on nosocomial BSI events occurring at least 48 h after ICU admission, which could explain the absence of a clear PSP increase before infection onset in our study. Moreover, the type of infection may lead to a different PSP response as our study focused on BSI compared to pneumonia, wound infections, and urinary tract infections in the study of Klein and colleagues. As such, the different findings may have resulted from variations in study design and infection characteristics [23].

A previous study conducted in critically ill adults admitted to the ICU reported serial increases in PSP levels prior to the onset of ICU-acquired sepsis [3]. In that study, there was an association between the diagnosis of clinical sepsis and an elevation in PSP levels during the days prior to diagnosis. Interestingly, unlike CRP (day 2) and procalcitonin (day 3), PSP exhibited an upward trend beginning as early as five days before the clinical diagnosis. In contrast, we measured daily PSP levels from 72 h before the occurrence of BSI events until BSI onset. Therefore, we cannot dismiss the possibility that a rise in PSP levels may have already occurred at an earlier time point. However, from a clinical perspective, a biomarker signal that appears more than 72 h in advance of BSI onset would not be very useful as it would not lead to actionable interventions.

We observed notably higher plasma PSP concentrations among patients who were more severely ill, suggesting that this biomarker could have prognostic value. In a previous study examining PSP trajectories in 83 critically injured patients, plasma levels were significantly higher in trauma patients who later developed sepsis compared to those having an uncomplicated infection in the ICU (with a median of 146 ng/mL versus 111 ng/mL, respectively) [9]. Furthermore, in that study patients admitted with higher severity of illness, as indicated by Acute Physiology and Chronic Health Evaluation (APACHE) scores of 17.8 ± 7.4, had higher PSP levels at the onset of infection compared to those with APACHE scores of 14.9 ± 7.2. In another study that, focused on sepsis patients requiring ICU admission, higher APACHE and Simplified Acute Physiology Score (SAPS) scores were also associated with elevated PSP concentrations upon ICU admission [24]. Similar differences in PSP levels were observed when comparing patients with septic shock to those with sepsis. These collective findings suggest a robust connection between PSP plasma concentration and general indicators of disease severity in critically ill patients, highlighting its potential prognostic significance.

Our study was performed in a tertiary care setting, with a patient population characterized by complex medical conditions and a high burden of organ dysfunction. The generally (very) high PSP concentrations in plasma that were observed in our study can likely be attributed to this. Notably, much lower PSP values have been observed in patients who were suspected of sepsis in a primary care setting (median PSP level 156 [90–286] vs. 131 [83–205] ng/ml for patients with and without sepsis, respectively) [25]. However, also in that study, the diagnostic utility of this biomarker seemed limited, as evidenced by a C-statistic of 0.57 (95% CI 0.49–0.63).

Our study has several strengths. First, the large MARS biorepository enabled us to analyse precisely selected ICU-acquired BSI events. This minimizes the risk of information bias. Furthermore, we used a matched-event analysis which resulted in pathogen groups with highly comparable clinical patient and event characteristics while preserving sufficient statistical power to assess relevant differences in PSP response between groups. Given that critically ill patients can be very heterogeneous, matching theoretically reduces unwanted variation due to extraneous factors [26].

Our study also has some limitations. First, we did not compare individual ICU patients with BSI to control patients without (ever) BSI but rather performed a within-event comparison of PSP concentrations before and after the onset of infection. Although this eliminates the need to adjust for differences between individuals, it does assume that study subjects did not already have BSI at baseline (T-72). To this end, we selected patients who did not have a positive blood culture in the 30 days prior to the included event. However, as blood cultures were not drawn every day, we cannot fully exclude the possibility of BSI at the first time point of study (i.e., 72 h before the first detection of the BSI event). As such, misclassification of T0 may have occurred, but we consider it highly unlikely that this would affect the majority of episodes. We, therefore, performed a sensitivity analysis in which we realigned PSP timelines with peak CRP levels, which did not change interpretation. We did not study a comprehensive sample of BSI events but deliberately restricted our analysis to infections caused by one of three specific pathogen types. This decision aimed to minimize the risk of unintentionally including contaminated blood draws. However, it does limit the generalizability of our findings to some extent. Finally, 48% of patients in our study had renal replacement therapy at baseline, which may have affected PSP kinetics. Considering that PSP is a 16 kDa protein it is likely subject to (some) elimination by this. Against this background, it will thus be more difficult to accurately assess the diagnostic value of this biomarker.

## Conclusions

We measured PSP concentrations over four consecutive days during the onset of ICU-acquired bloodstream infection due to commonly observed pathogens in critically ill patients. Overall, plasma levels were typically 3–7 times higher than the normal value and correlated with disease severity. However, we did not observe significant differences between pathogen groups, suggesting that PSP responses are similar in both bacterial (GNR and enterococci) and fungal (*Candida*) infections. No patterns in the PSP response over time were identified, thereby limiting the potential of PSP as an early diagnostic marker in the 3 days before BSI in ICU patients.

## Data Availability

The datasets used and/or analysed during the current study are available from the corresponding author on reasonable request.

## Abbreviations

ANOVA: Analysis of variance
APACHE: Acute Physiology and Chronic Health Evaluation
BSI: Bloodstream infection
CI: Confidence interval
CE: Conformité européenne
CRP: C-reactive protein
GNR: Gram-negative rods
ICU: Intensive care unit
IQR: Interquartile range
PSP: Pancreatic stone protein
SAPS: Simplified Acute Physiology Score
SOFA: Sequential Organ Failure Score
